# High-Thermal Stable Epoxy Resin through Blending Nanoarchitectonics with Double-Decker-Shaped Polyhedral Silsesquioxane-Functionalized Benzoxazine Derivatives

**DOI:** 10.3390/polym16010112

**Published:** 2023-12-29

**Authors:** Yang-Chin Kao, Jing-Yu Lin, Wei-Cheng Chen, Mohamed Gamal Mohamed, Chih-Feng Huang, Jung-Hui Chen, Shiao-Wei Kuo

**Affiliations:** 1 Department of Materials and Optoelectronic Science, Center for Functional Polymers and Supramolecular Materials, National Sun Yat-Sen University, Kaohsiung 80424, Taiwan; d123100002@nsysu.edu.tw (Y.-C.K.); chwei566@gmail.com (W.-C.C.); mgamal.eldin34@gmail.com (M.G.M.); 2 Department of Chemistry, National Kaohsiung Normal University, Kaohsiung 80201, Taiwan; sammy870703@gmail.com; 3 Chemistry Department, Faculty of Science, Assiut University, Assiut 71515, Egypt; 4 Department of Chemical Engineering, i-Center for Advanced Science and Technology (iCAST), National Chung Hsing University, Taichung 40227, Taiwan; huangcf@dragon.nchu.edu.tw; 5 Department of Medicinal and Applied Chemistry, Kaohsiung Medical University, Kaohsiung 80708, Taiwan

**Keywords:** epoxy, benzoxazine, DDSQ, thermal ring polymerization, nanocomposites, thermal stability

## Abstract

A series of di-functional benzoxazine (BZ) monomers was synthesized, specifically the double-decker silsesquioxane (DDSQ) cage structure (DDSQ-BZ). Comparative analyses were conducted between DDSQ-BZ monomers and the most commonly utilized bisphenol A-functionalized bifunctional benzoxazine (BPA-BZ) monomer. DDSQ-BZ compounds possess better thermal properties such as high char yield and high thermal decomposition temperature (*T*_d10_) after thermal ring-opening polymerization (ROP) because the inorganic DDSQ cage nanostructure features a nano-reinforcement effect. In addition, blending inorganic DDSQ-BZ compounds with epoxy resin was explored to form organic/inorganic hybrids with enhanced thermal and mechanical properties following thermal ROP. The improvement in mechanical properties is primarily attributed to the network structure formed by the cross-linking between DDSQ-BZ and the epoxy resin during thermal ROP, as well as hydrogen bonding interactions formed between the hydroxyl groups generated during thermal ROP and the Si-O-Si bonds in the DDSQ structure.

## 1. Introduction

Benzoxazines (BZ) are a class of compounds characterized by the benzene structure linked to a six-membered ring structure containing oxygen and nitrogen. Holly and Cope initially proposed the concept of a benzoxazine ring [1]. The benzoxazine synthesis involves the Mannich condensation reaction, which occurs between o-hydroxybenzylamine and formaldehyde, forming 3,4-dihydro-2H-1,3-benzoxazine. To synthesize the benzoxazine monomer, phenols are reacted with primary amine derivatives in an aqueous solution containing formaldehyde or formaldehyde derivatives, under specific environmental conditions via the Mannich condensation reaction [2,3,4,5,6,7,8,9,10,11,12,13,14,15,16,17].

Epoxy resins can be achieved through two methods: one involves the reaction of alkenes with peroxyacids and their derivatives, while the other embraces the addition reaction of alkenes with halogens, followed by the removal of halide salts utilizing a strong base, culminating in organic compounds featuring epoxy groups [18,19,20,21,22]. Epoxy polymerizations occur by utilizing suitable curing agents, such as acids or acid anhydrides (e.g., organic acids, phosphoric acid, and pyromellitic dianhydride) [23,24,25,26,27,28,29,30]. Alternatively, alcohols could also be added to epoxy monomers along with the appropriate catalysts, such as inorganic strong acids, Lewis acids, or sodium tert-butoxide. A successful epoxy polymerization results in the formation of a three-dimensional covalent network structure, leading to the desired hardening effect [30,31,32,33,34]. The toughness of epoxy resins can be improved via inter-molecular interactions between the thermosetting polymers (PBZ) and the O-H functional groups [35,36,37,38,39] that occur after carrying out the polymerization. POSS hybrids mixed into a polymer can be categorized as end-chain or side-chain varieties and created using mono-functional or multi-functional POSS nanomaterials [40,41,42,43,44,45,46]. The latter leads to the formation of insoluble crosslinking structures. Moreover, the addition of inorganic POSS nanomaterials to BZ/epoxy hybrids leads to enhanced oxidation resistance, improved thermal stability, and ameliorated mechanical properties [47]. 

In our work, epoxy resin and BZ resin were combined and formed a crosslinked structure. The aim was to compensate for the insufficient heat resistance of epoxy resin and improve its mechanical properties by taking advantage of the excellent thermal stability and higher glass transition temperature (*T*_g_) of BZ resin. In addition to using basic BZ resin, we also modified the BZ resin itself to obtain different properties [48,49,50,51]. For instance, we synthesized BZ resin using different primary amines (aniline and allylamine) to obtain two types of BZ resin with different functional groups [52] to investigate whether the different functional groups would have an impact on the polymerization of epoxy resin. Furthermore, inorganic nanoparticles were introduced into the BZ/epoxy hybrids using a polyhedral oligomeric silsesquioxane (POSS) with a *T*_10_ structure (the POSS cage consists of 10 silicon (Si) atoms) called double-decker silsesquioxane (DDSQ). After incorporation, an organic/inorganic nanocomposite was formed by blending the inorganic nanostructure of DDSQ with epoxy resin. The aim was to achieve a nano-reinforcement effect on the polymer by the inorganic nanostructure of DDSQ, to enhance the heat resistance and mechanical properties [53,54,55,56]. 

## 2. Materials and Methods

### 2.1. Materials

Bisphenol A and magnesium sulfate (MgSO_4_) were purchased from Alfa-Aesar. Ethyl acetate, 1,4-dioxane, aniline, allylamine, and paraformaldehyde were purchased from Sigma-Aldrich. The synthesis method for DDSQ-BZ was previously described [57]. The epoxy resin (BADGE) was obtained from Dow Chemical, with an epoxy equivalent weight (EEW) of 190 g/eq.

### 2.2. Synthesis of Bisphenol A Type of Benzoxazine (AN and AL)

A round-bottom flask fitted with a reflux condenser was used to combine bisphenol A (2.51 g, 10.99 mmol) and paraformaldehyde (1.32 g, 44.0 mmol). To this flask, 1,4-dioxane (50 mL) was added. The system was purged with nitrogen gas for three cycles, ensuring an inert atmosphere. Then, either aniline (4.10 g, 44.03 mmol) or allylamine (2.52 g, 44.14 mmol) was introduced. The resulting mixture was heated at 115 °C for 48 h. After cooling, gravity filtration was performed to separate the mixture. The solvent was removed using rotary evaporation, and the remaining residue was dried in a vacuum oven. Using aniline yielded a pale-yellow paste-like solid form of Bisphenol A Benzoxazine Aniline (AN) (4.81 g, 95%). On the other hand, employing allylamine produced an orange viscous liquid form of Bisphenol A Benzoxazine allylamine (AL) (3.98 g, 93%).

### 2.3. The Preparation of Benzoxazine/Epoxy Hybrids

Four types of hybrids were prepared by mixing BPA-BZ (AN and AL) with epoxy resin and DDSQ-BZ (DQAN and DQAL) with epoxy resin. AN and AL were mixed with epoxy at a weight ratio of 1/5, while DQAN and DQAL were mixed with epoxy at a weight ratio of 1/1, 1/3, and 1/5. The DDSQ-BZ fraction was stirred at 60 °C for 48 h, and the BPA-BZ fraction was stirred at 60 °C for 48 h, as presented in Figure S1. The resulting samples were AN/EP = 1/5 and AL/EP = 1/5 obtained from the mixture of AN and AL, and DQAN/EP = 1/1 (1/3, 1/5) and DQAL/EP = 1/1 (1/3, 1/5) obtained from the mixture of DQAN and DQAL. Each sample was placed in an aluminum pan and thermally polymerized sequentially at temperatures of 150, 180, 210, 240, and 270 °C for 2 h each. This process resulted in the formation of an epoxy copolymer with a dark-brown color. 

## 3. Results and Discussion

### 3.1. Synthesis of Bisphenol A-Functionalized Benzoxazine Monomer (BPA-BZ)

**Figure 1:**
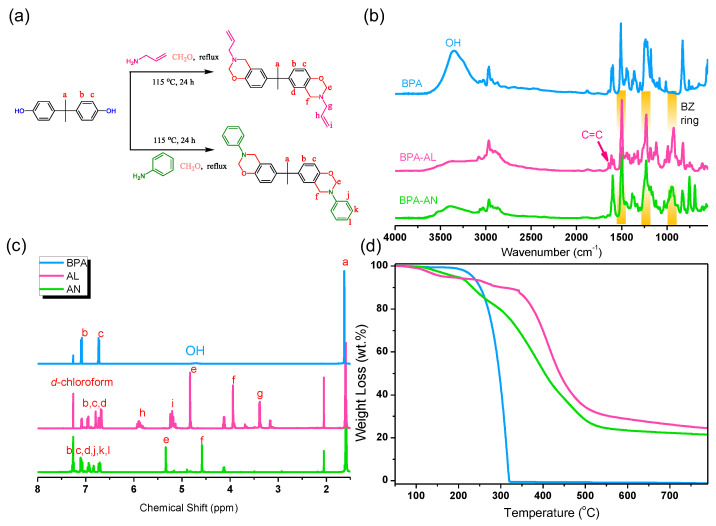
(**a**) Synthesis of AN and AL, (**b**) FT-IR spectrum, (**c**) ^1^H NMR spectrum, and (**d**) TGA curve.

Figure 1a illustrates the reaction process and structural representation of two BZ resins, AN and AL, synthesized through Mannich condensation reaction using bisphenol A (BPA), aniline, allylamine, and paraformaldehyde. To verify the synthesis of BZ resins, Fourier-Transform Infrared (FT-IR) spectroscopy, ^1^H Nuclear Magnetic Resonance (NMR) analysis, and thermogravimetric analysis (TGA) were employed to confirm the structures and investigate the thermal properties (Figure 1b–d). In the FTIR spectra of AN and AL, distinct signals were observed at 950 and 927 cm^−1^, corresponding to the absorption spectra of the oxazine ring, a characteristic feature of the BZ resin structure. Furthermore, peaks were observed at 1498 cm^−1^ and 1230 cm^−1^, representing the trisubstituted benzene and C-O-C bond structures in the BZ resin, respectively. Noteworthily, the spectrum of AL displayed a peak at 1644 cm^−1^, indicative of the characteristic absorption of the C=C double bond in the allyl functional group. Comparing AN and AL to the original BPA spectrum, a significant decrease in intensity was observed, specifically in the signal associated with the OH functional group, customarily found between 3100 cm^−1^ and 3500 cm^−1^. The disappearance of the OH signal indicates the completion of the substitution reaction and the formation of the BZ resin. By analyzing the ^1^H NMR spectra shown in Figure 1c, the structural signals of BPA, AL, and AN can be identified and matched with the labels indicated in Figure 1a. These signals encompass the aliphatic portion of BPA (designated as *a* at 1.62 ppm) and the aromatic signals (identified as *b* at 7.08 ppm and *c* at 6.74 ppm). The broad signal at 4.71 ppm corresponds to the OH group. After analyzing AN and AL, it was observed that the signal for the OH group in the BPA spectrum disappeared, and new signals representing the BZ cyclic structure appeared. In the case of AN, the identified signals consist of *e* (5.54 ppm), representing the OCH_2_N structure, and *f* (4.58 ppm), representing ArCH_2_N. For AL, the signals include *e* (4.83 ppm), representing the OCH_2_N structure, and *f* (3.94 ppm), representing ArCH_2_N. The introduction of the BZ resin structure resulted in a slight modification of the signal corresponding to the aromatic portion (labeled as *d*). Additionally, the functional groups that differentiate the two BZ resins exhibit distinctive signals. In the case of AN, the signals for the phenyl group originating from aniline are labeled as *j*, *k*, and *l*, and they coincide with the aromatic signals of the BPA structure. In AL, the signals representing the allyl functional group are labeled as *i* (5.20 ppm) and *h* (5.89 ppm) in a ratio of 2/1. The aliphatic signal connecting the nitrogen atom and the allyl group is labeled as *g* (3.38 ppm). The thermal stability of the synthesized AN and AL monomers was subsequently verified using TGA analysis, as depicted in Figure 1d. Both AN and AL exhibit significant enhancements in char yield compared to BPA. This enhancement in thermal stability can be attributed to the capability of the BZ resin monomers to undergo polymerization, resulting in the formation of a three-dimensional network structure at higher temperatures. This network structure acts as a protective layer, preventing contact with air and promoting the formation of char residue.

### 3.2. Synthesis of Double-Decker Silsesquioxane-Functionalized Benzoxazine Monomer (DDSQ-BZ)

**Figure 2:**
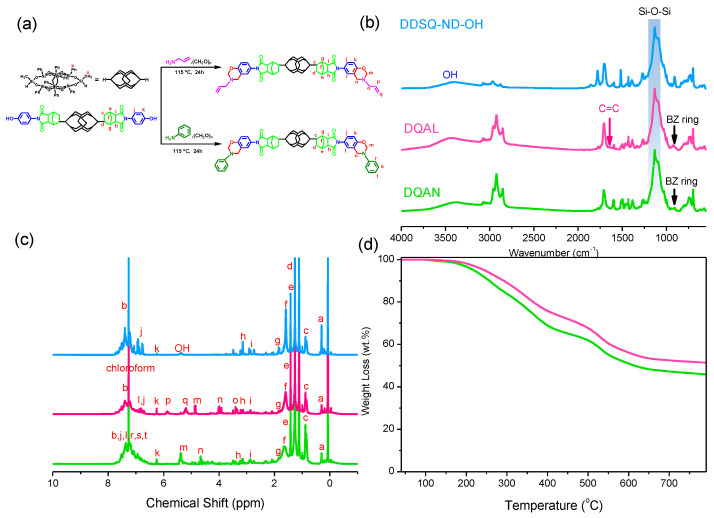
(**a**) Synthesis of DQAN and DQAL, (**b**) FT-IR, (**c**) ^1^H NMR spectrum, and (**d**) TGA curve.

In Figure 2a, the structure of DDSQ is depicted, along with the synthesis process and structural representation of two BZ resins, named DQAN and DQAL, respectively. These resins were synthesized by replacing BPA, aniline, allylamine, and paraformaldehyde with DDSQ-ND-OH, which incorporates the DDSQ structure, through a Mannich condensation reaction. Similarly, to verify the successful preparation of the BZ resins, FT-IR spectroscopy and ^1^H NMR analysis were performed to confirm the accuracy of the compositional changes in the structures, and TGA was conducted to examine the thermal properties of the two products (Figure 2b–d). By examining the FT-IR spectral signals presented in Figure 2b, distinct peaks corresponding to the DDSQ structure can be observed in the spectra of DDSQ-ND-OH, DQAN, and DQAL. These peaks manifest at 1132 cm^−1^ and 1272 cm^−1^, representing the Si-O-Si bond and Si-CH_3_ absorption, respectively. In the DDSQ-ND-OH spectrum, stretching signals of the C=O bond are observed at 1702 cm^−1^ and 1780 cm^−1^, while a signal at 1390 cm^−1^ indicates the stretching of the C-N bond. Additionally, a broad signal centered around 3420 cm^−1^ is observed, indicating the characteristic peak of the hydroxyl (OH) functional group in the phenolic structure. This functional group replaces the phenolic part of BPA during the synthesis of the BZ resin. After the completion of the condensation reaction, the spectra of DQAN and DQAL demonstrate a decrease in the characteristic peaks attributed to the OH group in DDSQ-ND-OH. Simultaneously, distinctive signals indicating the trisubstituted benzene and C-O-C bond structures emerge at approximately 1500 cm^−1^ and 1234 cm^−1^, respectively. The signals corresponding to the BZ ring exhibit similarities to the BZ ring signals observed in the AN and AL monomers shown in Figure 1b, appearing around 943 cm^−1^ (DQAN) and 923 cm^−1^ (DQAL). In Figure 2c, the corresponding ^1^H NMR spectra of the three compounds are analyzed and matched with the indicated labels in Figure 2a. The signal labeled *a* at 0.37 ppm corresponds to the Si-CH_3_ signal in the DDSQ structure, while the signal labeled *b* represents the aromatic part of the DDSQ structure, distributed in the range of 7.11–7.48 ppm. The alkyl signals of the ND anhydride structure in *DDSQ-ND-OH* are located between 0.88 ppm and 3.20 ppm, labeled as *h* (3.20–3.25 ppm), *i* (2.90 ppm), *e*–*g* (1.35–1.42 ppm), *d* (1.25 ppm), and *c* (0.85 ppm). In the DDSQ-ND-OH spectrum, a broad signal indicative of the phenolic structure, equivalent to the OH groups in BPA, is detected at 5.35 ppm. Additionally, signals representing the aromatic portion are observed at positions labeled as *j* (6.80 ppm) and *k* (6.24 ppm). The disappearance of OH group signals and the appearance of BZ ring signals confirm the presence of the synthesized BZ resin. In DQAN, these signals are found at 5.33 ppm (labeled as *m*, OCH_2_N) and 4.65 ppm (labeled as *n*, ArCH_2_N), while in DQAL, they appear at 4.85 ppm (labeled as *e*) and 3.98 ppm (labeled as *f*). Similar to AN and AL, different functional groups give rise to distinct characteristic peaks. In DQAN, with a benzene structure, its signals overlap with other aromatic signals (labeled as *r*, *s*, *t*), whereas in DQAL, with an alkene structure, the signals representing the alkene functional group are labeled as *i* (5.20 ppm) and *h* (5.89 ppm) in a 2/1 ratio. The signal representing the alkyl group connecting the nitrogen atom and the alkene is observed at 3.38 ppm (labeled as *o*). Finally, as shown in Figure 2d, both DQAN and DQAL exhibited improved thermal stability compared to the BPA-BZ resin, particularly evident in the significant increase in char yield. This enhancement can be attributed to the introduction of the DDSQ structure, an inorganic POSS nanomaterial containing numerous inorganic Si-O-Si bonds. These bonds are not vaporized or lost during high-temperature annealing and, when combined with the network structure formed by the opening of the BZ resin, result in an increased residue of carbonaceous char, thereby enhancing its thermal performance.

### 3.3. Thermal Polymerization of BPA-BZ Monomer and Epoxy Resin

**Scheme 1:**
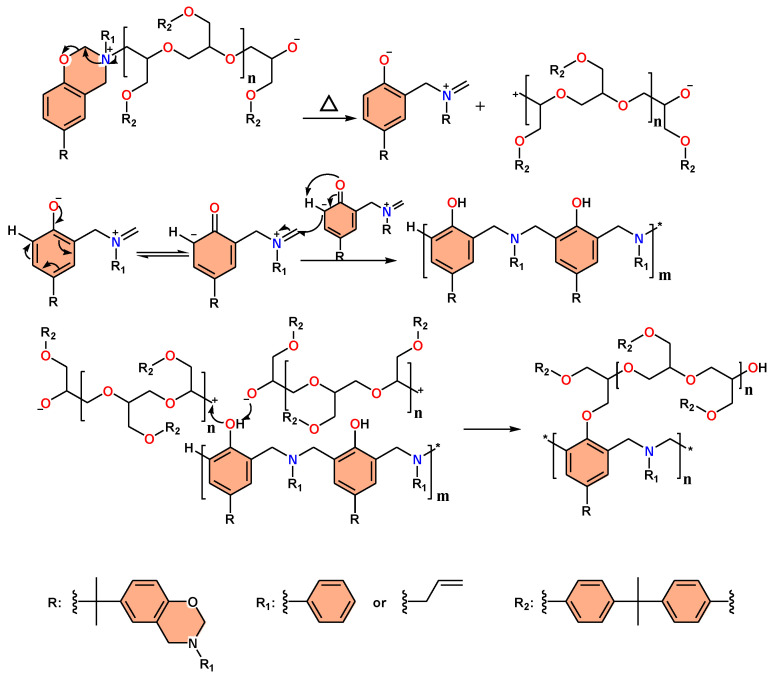
The reaction mechanism of the polymerization of BZ resin that reacts with epoxy resin to promote the ring opening of epoxy resin.

**Figure 3:**
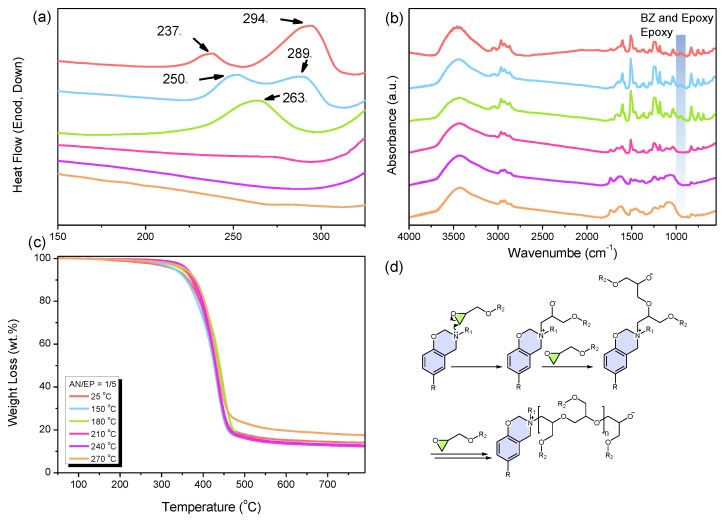
(**a**) DSC curves of AN/EP = 1/5, measured after heat treatment at room temperature 25 °C and 150, 180, 210, 240, and 270 °C, (**b**) FT-IR spectrum, (**c**) TGA curve, and (**d**) reaction mechanism of epoxy resin polymerization promoted by BZ resin.

The AN and AL monomers of BPA-BZ were blended with epoxy resin in a specific ratio, followed by sequential thermal treatments (25, 150, 180, 210, 240, and 270 °C) to induce crosslinking. The properties of the polymer blends before and after crosslinking (at 25 and 270 °C) and during the crosslinking process were analyzed using DSC, FT-IR spectroscopy, and TGA analysis. Figure 3a visually represents the blending process of AN with epoxy resin in a weight ratio of 1/5, resulting in a polymer blend denoted as AN/EP = 1/5. For room temperature curing (25 °C), two distinct exothermic peaks are observed in DSC spectra shown in Figure 3a. The first exothermic peak at 237 °C signifies heat release from the reaction between the epoxy resin and BZ resin, initiating their respective polymerization processes [58]. The peak at 294 °C indicates the heat release resulting from the ring-opening polymerization of the BZ monomers, which promotes the polymerization of the epoxy resin at higher temperatures. The reaction mechanism of the self-polymerization of the epoxy resin and BZ resin is depicted in Figure 3d, while the polymerization mechanism of the BZ resin reacting with the epoxy resin is shown in Scheme 1. 

A slightly higher temperature is required with a BZ resin epoxy blend compared to pure BZ resin polymerization because the high concentration of epoxy resin dilutes the crosslinking of the BZ resin. Furthermore, the crosslinking of the epoxy resin restricts the mobility of the BZ monomer, leading to a higher polymerization temperature. The exothermic peaks gradually decrease in intensity and shift towards higher temperatures at higher reaction temperatures. This observation signifies the successful polymerization. These findings can be further supported by referring to the FT-IR spectra under different thermal treatment conditions, as shown in Figure 3b. The characteristic peaks around 950 cm^−1^, representing the BZ ring of BZ resin and the epoxy functional group of epoxy resin, gradually decrease and eventually vanish during the thermal treatment process. In Figure 3b, the absorption peak of Si-O-Si is observed at 1242 cm^−1^ for the AN/EP sample at 25 °C. Upon elevating the thermal curing temperature from 150 to 270 °C, the absorption band of Si-O-Si shifts to 1232 cm^−1^. This shift suggests the presence of hydrogen bonding interactions between AN and EP, highlighting the dynamic changes in the Si-O-Si absorption peak as a function of temperature. Figure 3a,b confirm that after the 210 °C thermal treatment, the majority of the peaks vanished, indicating that polymerization is almost complete. Results of TGA measurements on the materials polymerized at different temperatures are presented in Figure 3c. As the temperature increases, there is a noticeable enhancement in *T*_d10_ and char yield indicating polymerization, resulting in the formation of a network structure with enhanced thermal stability. These findings correspond to the gradual disappearance of reactants observed in Figure 3a,b. 

**Figure 4:**
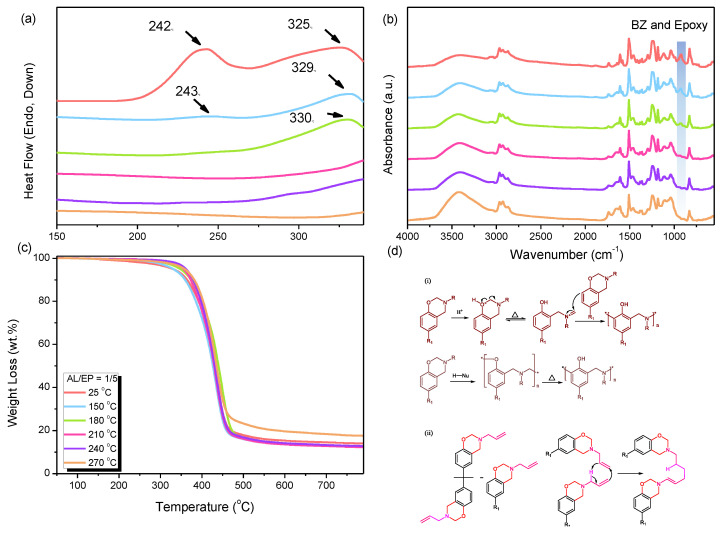
(**a**) DSC curves of AL/EP = 1/5, measured after heat treatment at room temperature 25 °C and 150, 180, 210, 240, and 270 °C, (**b**) FT-IR spectrum, (**c**) TGA curve and (**d**) (**i**,**ii**) presents thermal curing reaction of BZ ring and C=C double bond in BZ resin.

The same analysis can also be applied to similar AL and epoxy resin polymer blends. Following the same experimental procedure used for preparing the AN polymer blend, the AL and epoxy resin, both BZ resins, were blended in a weight ratio of 1/5 to obtain the polymer blend. This polymer blend is referred to as AL/EP = 1/5. Similarly, it underwent sequential thermal treatments starting from room temperature (25 °C) and gradually increasing to 150, 180, 210, 240, and 270 °C, respectively. Figure 4a illustrates the findings of the DSC analysis conducted under the same measurement conditions as the AN polymer blend. The orange curve, which represents the untreated sample at 25 °C, closely resembles the curve of AN/EP = 1/5 at 25 °C. Both curves exhibit two distinct exothermic peaks; however, there are slight discrepancies. In the case of AL/EP = 1/5, the first exothermic peak emerges at 242 °C. This peak signifies the exothermic reaction resulting from the combination of epoxy resin with BZ resin, thereby promoting the polymerization reaction between epoxy resin molecules. Furthermore, this peak also encompasses the exothermic peak generated during the thermal curing of the alkene groups (C=C double bonds) in AL [59]. The reaction mechanism for the alkene C=C double bond curing is depicted in Figure 4d. The second exothermic peak, occurring at 325 °C, is identical to that of AN/EP = 1/5 and is generated by the completely ring-opened benzoxazine. Similarities between AL/EP = 1/5 and AN/EP = 1/5 include the dilution effect caused by a substantial amount of epoxy resin in the polymer blend and the restricted mobility resulting from the polymerization of the epoxy resin. Also, the temperature at which the exothermic peak of the BZ resin with epoxy is slightly elevated compared to pure BZ resin polymerization, and the exothermic peaks gradually weaken during the thermal treatment steps. Furthermore, the presence of C=C double bonds in the AL/EP (allyl/epoxy) blend at a ratio of 1/5 has a significant impact on the mobility of the polymer blend during thermal curing. This effect contributes to an increase in the polymerization temperature of the benzoxazine (BZ) resin. When comparing the FT-IR spectra for different thermal treatment conditions to the reference spectrum shown in Figure 4b, a similar trend to the AN/EP (aniline/epoxy) blend at a ratio of 1/5 can be observed. It is evident that distinctive peaks associated with the benzoxazine ring of the BZ resin and the epoxy functional group of the epoxy resin, located around 923 cm^−1^, gradually diminish and eventually disappear during the thermal treatment process. The absence of these peaks after thermal treatment at 210, 240, and 270 °C indicates a significant advancement in the polymerization process. A notable difference in the DSC analysis is the absence of the peak associated with the curing of the BZ ring after thermal treatment at 180 °C, indicating the completion of the curing process. Furthermore, Figure 4c presents an assessment of the thermal properties of the AL/EP = 1/5 blend under various thermal treatment conditions. The parameters used for this evaluation are the 10% weight loss temperature (T*_d_*_10_) and char yield, which serve as indicators of thermal stability. The results demonstrate that as the thermal treatment progresses and reaches 270 °C, the AL/EP = 1/5 blend exhibits improvements in the values of T*_d_*_10_ and char yield. These enhancements indicate the occurrence of polymerization reactions and the formation of a highly thermally stable network structure. These findings corroborate the observations made in the DSC and FT-IR analyses.

### 3.4. Thermal Polymerization of DDSQ-BZ Monomer and Epoxy Resin

**Figure 5:**
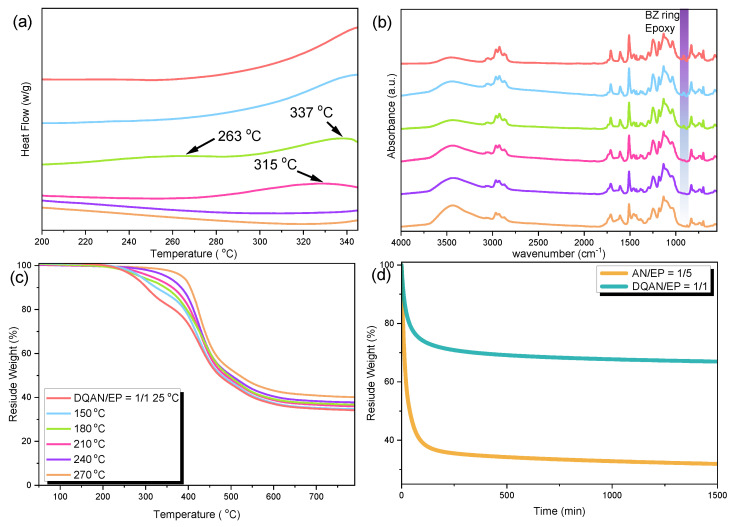
(**a**) DSC curves of DQAN/EP = 1/1, measured after heat treatment at room temperature 25 °C and 150, 180, 210, 240, and 270 °C, (**b**) FT-IR spectrum, (**c**) TGA curve and (**d**) aging test of DQAN/EP = 1/1 with AN/EP = 1/5 (recorded at 380 °C).

After analyzing two different variants of BZ resins that contain the bisphenol A (BPA) structure, the investigation moved forward to evaluate copolymer blends of DQAN and DQAL. These blends integrated the DDSQ structure as a substitute for the BPA component in the BPA-BZ resin. To replicate the reaction conditions used for the copolymerization of BPA-BZ, DQAN and DQAL were mixed with epoxy resin in a 1/1 weight ratio, mirroring the weight ratio employed in the BPA-BZ copolymerization. Following this, the same thermal treatment steps were conducted at temperatures of 25, 150, 180, 210, 240, and 270 °C to produce a highly thermally stable network structure. The properties of the samples were assessed before and after the crosslinking process using DSC analysis, FT-IR spectroscopy, and TGA analysis. Figure 5 provides an analysis of DQAN/EP with a 1/1 ratio under various thermal treatment conditions. The DSC analysis results, as depicted in Figure 5a, show that the orange line in the graph represents the sample at room temperature (25 °C) without any thermal treatment. It displayed an exothermic peak signal spanning the range of 250 to 350 °C, with a less pronounced exothermic peak occurring around 243 °C. As observed in the previously discussed copolymer blends (BPA-BZ/EP), the faint exothermic peak at 243 °C indicated exothermic signals arising from the self-polymerization of the epoxy resin when combined with the BZ resin. The distinct exothermic peak signal between 250 and 350 °C signified a substantial release of heat attributed to significant decomposition. This peak, corroborated by the TGA analysis of the uncuring DQAN/EP = 1/1 in Figure 5c, corresponded to a significant weight loss around 250 °C, persisting until approximately 360 °C. Following exposure to thermal treatment at 180 °C, the exothermic peak of DQAN/EP = 1/1 exhibited a split into two components: the epoxy resin ring opening occurred at 263 °C, while the BZ resin ring opening took place at 337 °C. This phenomenon resembled the situation observed with BPA-BZ. The combination of a substantial presence of epoxy resin and the limited mobility resulting from the crosslinking of epoxy resin created a notable dilution effect. Consequently, higher temperatures were required for the self-ring-opening polymerization of the BZ resin. Additionally, during the thermal curing process of DQAN/EP = 1/1 at 210 and 270 °C, the exothermic peak gradually diminished and shifted to higher temperatures. Comparing the results to the representative FT-IR spectrum in Figure 5b, similar to the data observed in the case of BPA-BZ, we can see that the characteristic peaks associated with the BZ ring and the epoxy functional group around 950 cm^−1^ exhibit a gradual decrease and eventually vanish as a result of the progressive thermal treatment. The TGA analysis curves depicted in Figure 5c illustrate the thermal characteristics of the DQAN/EP = 1:1 ratio under various heat treatments. To evaluate its thermal stability, we examined the temperatures corresponding to T*_d_*_10_ and the char yield. The findings reveal an increase in both the T*_d_*_10_ values and char yield for the DQAN/EP = 1/1 after thermal treatment. These results provide compelling evidence of polymerization reactions taking place and the formation of a thermally stable network structure. In the TGA analysis, an aging test was conducted for two different samples: AN/EP = 1/5 and DQAN/EP = 1/1. This test is illustrated in Figure 5d and involved heating at a rate of 20 °C per minute, starting from 50 °C and going up to 380 °C. Subsequently, the temperature was held constant at 380 °C for 1500 min. During this extended period, data were recorded and analyzed to assess thermal stability based on weight loss. The results unequivocally demonstrated that the DQAN/EP = 1/1 exhibited significantly higher mass residue and greater thermal stability when compared to the AN/EP = 1/5. 

**Figure 6:**
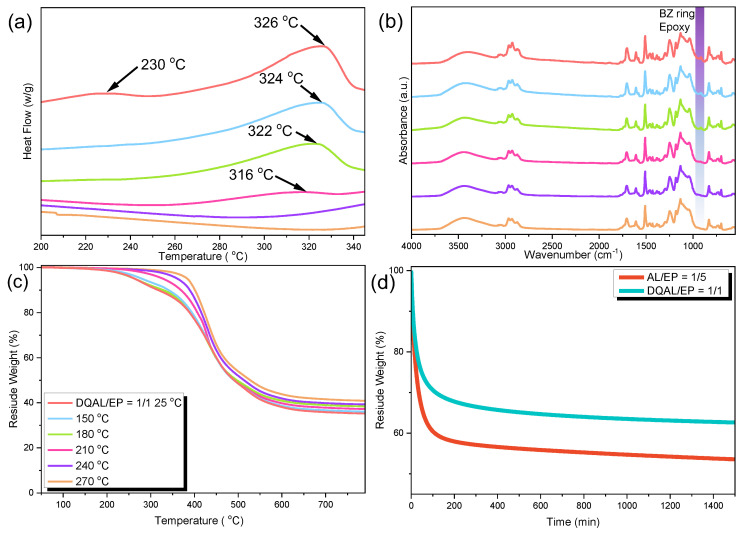
(**a**) DSC curves of DQAL/EP = 1/1, measured after heat treatment at room temperature 25 °C and 150, 180, 210, 240 and 270 °C, (**b**) FT-IR spectrum, (**c**) TGA curve and (**d**) aging test of DQAL/EP = 1/1 with AL/EP = 1/5 (recorded at 380 °C).

Next, DQAL was mixed with EP in a 1/1 weight ratio to create DQAL/EP = 1/1, and, subsequently, DQAL/EP = 1/1 underwent a series of thermal treatments, ranging from 25 °C to 270 °C, to produce crosslinked materials of DQAL/EP. The DSC analysis results for DQAL/EP = 1/1 during various thermal curing stages are depicted in Figure 6a. When examining the representative orange line, which corresponds to the uncured DQAL/EP sample at 25 °C, it displays similarities to the results obtained for AL/EP = 1/5. Both DQAL/EP and AL/EP samples exhibit two distinct exothermic peaks. The first exothermic peak of DQAL/EP = 1/5 is observed at 230 °C, signifying the heat release from the curing of double bonds (C=C) and the ring-opening polymerization of the EP resin. The second peak, occurring at 326 °C, represents the exothermic reaction resulting from the ring-opening polymerization of the BZ resin within DQAL. During the heat treatment process ranging from 150 °C to 270 °C, the exothermic peak associated with the ring-opening polymerization (ROP) of the oxazine unit in the BZ monomer within DQAL/EP gradually diminishes. This diminishing peak suggests that the ROP process of the oxazine unit has reached completion, indicating a successful and thorough polymerization reaction. The distinctive peak around 923 cm^−1^ in both the BZ ring and EP diminishes significantly and eventually vanishes entirely following thermal curing, as depicted in Figure 6b. As illustrated in Figure 6c, the values for T*_d_*_10_ and char yield of DQAL/EP exhibit a rise as the level of thermal treatment increases. This phenomenon can be attributed to the development of crosslinking structures originating from the combination of PBZ and EP. The results from the aging test conducted at 380 °C, as depicted in Figure 6d, clearly demonstrated that the DQAL/EP = 1/1 exhibited significantly greater residual mass and superior thermal stability in comparison to the AL/EP = 1/5.

### 3.5. Thermal and Mechanical Properties of DDSQ-BZ and Epoxy Hybrids

**Figure 7:**
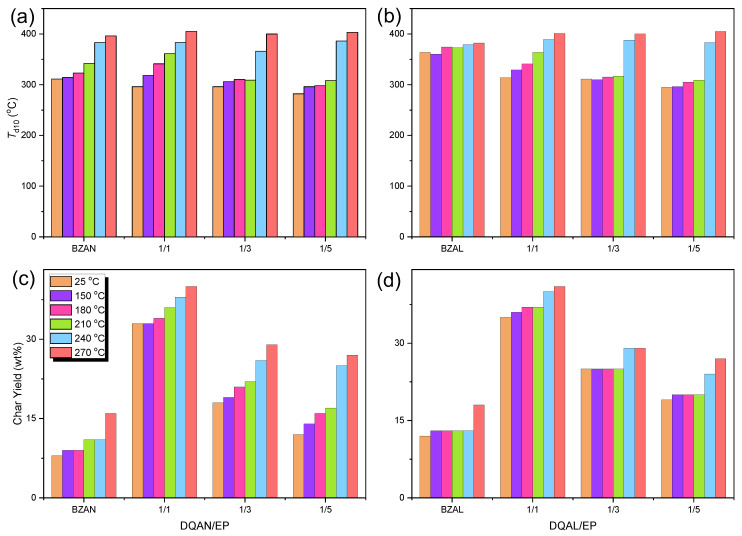
*T_d_*_10_ and char yield of each copolymer at different heat treatment stages (**a**,**c**) AN/EP = 1/5 and DQAN/EP = 1/1 to 1/5 and (**b**,**d**) AL/EP = 1/5 and AN/EP = 1/5 and (**d**) DQAL/EP = 1/1 to 1/5.

To illustrate the thermal properties of eight distinct polymer blends, we grouped them into two categories: BZ resin/epoxy resin blends and DQAN/DQAL resin/epoxy resin blends. These blends have varying ratios of their components (AL/EP or DQAN/DQAL to EP) to create a comprehensive dataset. The resulting graphical representation of their thermal characteristics, specifically focusing on parameters like T*_d_*_10_ (the temperature at which 10% mass loss occurs) and char yield under different heat treatment conditions, can be observed in Figure 7. Upon analyzing Figure 7a,b, it becomes evident that the inclusion of the DDSQ structure in the crosslinked polymer blends has a slightly favorable impact on T*_d_*_10_ when comparing similar molar ratios of AN/EP = 1/5 and DQAN/EP = 1/1, or AL/EP = 1/5 and DQAN/EP = 1/1. Similarly, the presence of the DDSQ structure in the BZ polymer blends also leads to a modest enhancement in T*_d_*_10_ when examining various ratios of DQAN/EP = 1/1 (1/2, 1/3) or DQAL/EP = 1/1 (1/2, 1/3). Focusing on Figure 7c,d, it is evident that the DDSQ structure has a significant influence on the char yield. It is clear that as the proportion of DDSQ-based BZ blends increases, the char yield proportionally increases as well. This rise in char yield can be mainly attributed to the substantial presence of nanoscale inorganic structures within DDSQ. These structures effectively safeguard the overall integrity of the polymer blends and result in the generation of more char residue. Consequently, it can be inferred that the incorporation of inorganic DDSQ nanoparticles into the structure substantially augments the thermal stability of BZ and epoxy resin polymer blends. 

**Figure 8:**
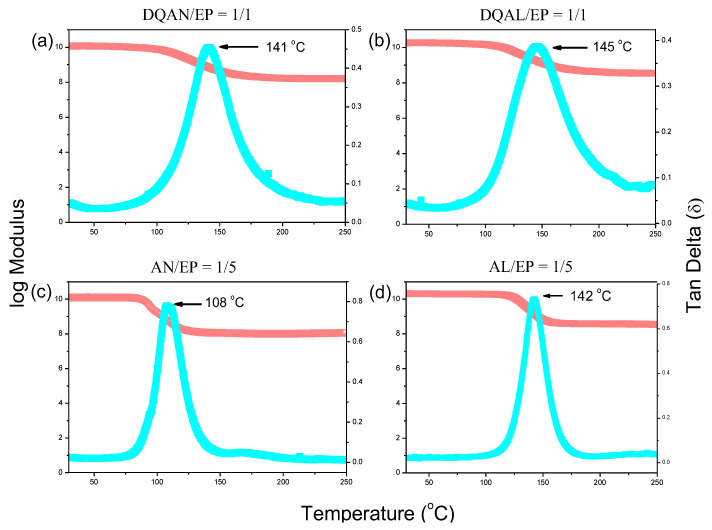
The blend of DDSQ-BZ resin and BPA-BZ resin with a similar molar ratio to epoxy resin (**a**) DQAN/EP = 1/1, (**b**) DQAL/EP = 1/1, (**c**) AN/EP = 1/5 and (**d**) DQAL/EP = 1/1 to 1/5 AL/EP = 1/5 DMA analysis chart after thermal polymerization.

Figure 8a–d compare the BZ resin/epoxy resin copolymers with different functional groups, namely AN/EP = 1/5, AL/EP = 1/5, DQAN/EP = 1/1, and DQAL/EP = 1/1. After undergoing thermal treatment up to 270 °C, the samples were subjected to dynamic mechanical analysis (DMA) to obtain relevant data on mechanical properties, such as storage modulus (*E*′), loss tangent (tan δ), and calculated glass transition temperature (*T_g_*). These data were used to compare the mechanical properties of the copolymers after polymerization. Firstly, the copolymers with a benzene functional group, AN/EP = 1/5 and DQAN/EP = 1/1, were compared. At 25 °C, DQAN/EP = 1/1 exhibited an initial storage modulus (*E*′) of 11,269 MPa and a *T_g_* of 142 °C, while AN/EP = 1/5 had an initial storage modulus of 12,007 MPa and a *T_g_* of 108 °C. In the second comparison, the copolymers with an acrylic functional group, AL/EP = 1/5 and DQAL/EP = 1/1, were compared. DQAL/EP = 1/1 showed an initial storage modulus (*E*′) of 17,503 MPa and a *T_g_* of 147 °C, whereas AL/EP = 1/5 had an initial storage modulus of 20,230 MPa and a *T_g_* of 141 °C. The data indicate that when the functional groups are the same and the molar ratio of the BZ resin to epoxy resin is similar, the inclusion of rigid inorganic DDSQ structures in the BZ resin significantly increases the *T_g_* of the copolymer while maintaining comparable mechanical properties. Thus, the introduction of DDSQ structures enhances thermal performance while preserving the mechanical properties. On the other hand, when comparing copolymers with different functional groups, it is evident that copolymers with a benzene functional group, such as AN/EP, exhibit significantly lower initial storage modulus and *T_g_* values compared to copolymers with an acrylic functional group, such as AL/EP. This difference arises from the acrylic functional group undergoing thermal curing through double bond conversion at high temperatures, leading to a higher crosslinking density and reduced flowability when combined with the polymerization of BZ resin and epoxy resin. The increased crosslinking density and reduced flowability contribute to improved thermal and mechanical properties. Consequently, compared to benzene-functionalized BZ resin, the changes in acrylic-functionalized BZ resin after the introduction of DDSQ structures are less pronounced. 

**Figure 9:**
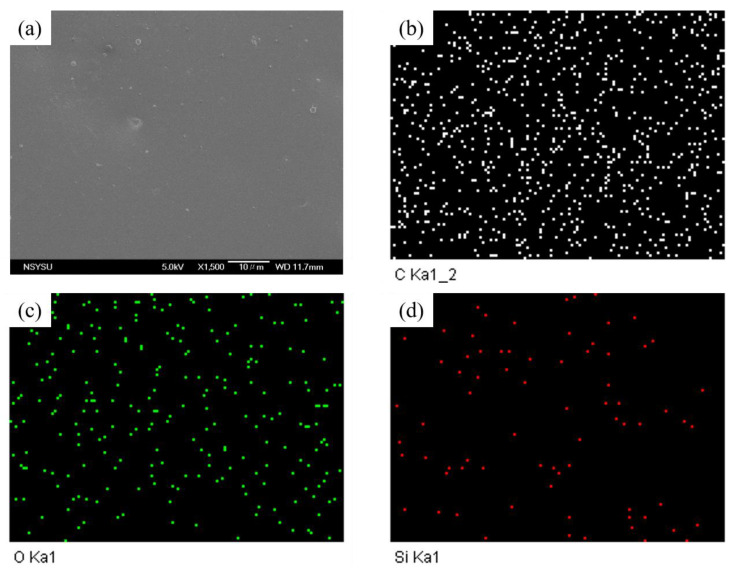
(**a**) SEM, (**b**) C-mapping, (**c**) O-mapping, and (**d**) Si mapping of DQAN/EP = 1/1 after thermal polymerization.

**Figure 10:**
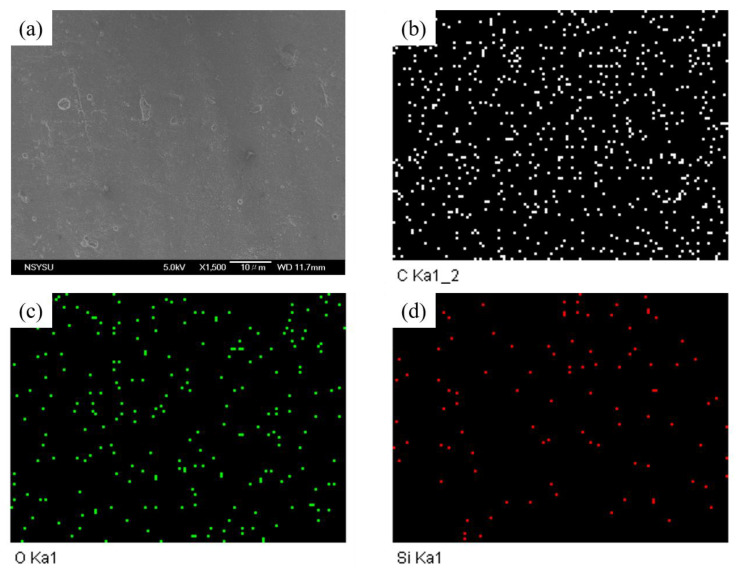
(**a**) SEM, (**b**) C-mapping, (**c**) O-mapping, and (**d**) Si-mapping of DQAL/EP = 1/1 after thermal polymerization.

To confirm the uniform dispersion of inorganic DDSQ cage-like structures in the BZ resin/epoxy resin copolymers, scanning electron microscopy (SEM) was employed. Figure 9a and Figure 10a show SEM images of the samples after completing all thermal treatments, representing DQAN/EP = 1/1 and DQAL/EP = 1/1, respectively. 

In both images, no distinct phase separation or characteristic morphology was observed, indicating the homogeneous dispersion of BZ resin with inorganic DDSQ nanoparticles during polymerization and the uniform crosslinking with the epoxy resin. Additionally, SEM analysis was used to map the elements C, O, and Si, confirming the atomic distribution and uniform dispersion of BZ resin and epoxy resin in the composition. The mapping results are presented in Figure 9b–d and Figure 10b–d, providing further evidence of the even dispersion of DDSQ particles within the epoxy resin.

## 4. Conclusions

We successfully synthesized BPA-BZ and DDSQ-BZ through Mannich condensation reactions. After undergoing a series of thermal treatments, the char yield of the DDSQ-BZ monomer was significantly higher than that of the commonly used typical BZ resin monomer, BPA-BZ (without DDSQ cage-like structures). This enhancement can be attributed to the inorganic DDSQ structures, which improve the thermal properties of the resin through a nano-reinforcement effect. SEM and TEM images confirmed the homogeneous distribution of the inorganic DDSQ cage-like bodies within the epoxy resin. Consequently, the *T*_g_ value, thermal properties, and storage modulus of the DDSQ-BZ/epoxy resin copolymer measured by DMA were enhanced. This improvement is primarily due to the hydrogen bonding interactions between the hydroxyl functional groups formed by the epoxy resin’s ring-opening thermal polymerization and the Si-O-Si bonds in the inorganic DDSQ cage-like bodies. These interactions restrict the mobility of the polymer chains (observable in FT-IR spectra). On the other hand, the hydroxyl functional groups generated by the BZ resin during thermal polymerization also participate in intramolecular hydrogen bonding. The combination of these two types of hydrogen bonding interactions and the covalent bonding between the resins contributes to the improved mechanical properties of the copolymer. Taking DQAN/EP = 1/1 as an example of DDSQ-BZ, the *T_g_* and *T_d_*_10_ values (141 °C and 405 °C, respectively, as determined by DMA and TGA) are higher than those of the equivalent organic hardener (BPA-BZ) when added to the epoxy resin DGEBA. This confirms the influence of the DDSQ cage-like inorganic nanomaterial on rigidity. This research will pave the way for investigating the potential applications of BZ and epoxy resin combined with DDSQ, leading to the creation of advanced materials tailored for high-performance applications.

## Data Availability

The data presented in this study are available on request from the corresponding author.

## Supplementary Materials

The following supporting information can be downloaded at: https://www.mdpi.com/article/10.3390/polym16010112/s1, Figure S1: Schematic cartoon for preparation of benzoxazine/epoxy hybrids.
