# Supplementary material for: High-Thermal Stable Epoxy Resin through Blending Nanoarchitectonics with Double-Decker-Shaped Polyhedral Silsesquioxane-Functionalized Benzoxazine Derivatives
Source: Polymers (Basel). 2023 Dec 29;16(1):112. doi: 10.3390/polym16010112 (PMC10780953; doi:10.3390/polym16010112)
Supplement: Supplementary file 1 [file polymers-16-00112-s001.zip › polymers-2745305-supplementary.pdf]

Supporting Information

High Thermal Stable Epoxy Resin Through Blending Nanoarchitectonics with Double-Decker–Shaped Polyhedral Silsesquioxanes-Functionalized Benzoxazine Derivatives

Yang-Chin Kao[1,#], Jing-Yu Lin[2,#], Wei-Cheng Chen[1], Mohamed Gamal Mohamed,[1,3] Chih-Feng Huang,[4] Jung-Hui Chen[2,*], and Shiao-Wei Kuo[1,5,*]

[1]Department of Materials and Optoelectronic Science, Center of Crystal Research and Center for Functional Polymers and Supramolecular Materials, National Sun Yat-Sen University, Kaohsiung 80424, Taiwan; d123100002@nsysu.edu.tw (Y.C.K.); chwei566@gmail. com (W.C.C.); mgamal.eldin34@gmail.com (M.G.M); and kuosw@faculty.nsysu.edu.tw (S.W.K).

[2]Department of Chemistry, National Kaohsiung Normal University, Kaohsiung 802, Taiwan; sammy870703@gmail.com (J.Y.L); t1446@mail.nknu.edu.tw (J.-H.C.)

[3]Chemistry Department, Faculty of Science, Assiut University, Assiut 71515, Egypt; mgamal.eldin34@gmail.com (M.G.M).

[4]Department of Chemical Engineering, i-Center for Advanced Science and Technology (iCAST), National Chung Hsing University, 145 Xingda Road, South District, Taichung 40227, Taiwan; HuangCF@dragon.nchu.edu.tw (C.F.H).

[5]Department of Medicinal and Applied Chemistry, Kaohsiung Medical University, Kaohsiung 807, Taiwan; kuosw@faculty.nsysu.edu.tw (S.-W.K)

# Equal contribution

Corresponding authors: E-mail: t1446@mail.nknu.edu.tw (J.-H.C) and kuosw@faculty.nsysu.edu.tw (S.-W.K)

Characterization

FTIR spectra were collected on a Bruker Tensor 27 FTIR spectrophotometer with a resolution of 4 cm^{-1} by using the KBr disk method. ^{13}C nuclear magnetic resonance (NMR) spectra were examined using an INOVA 500 instrument with DMSO-d_6 and $CDCl_3$ as the solvent and TMS as the external standard. Chemical shifts are reported in parts per million (ppm). The thermal stabilities of the samples were performed by using a TG Q-50 thermogravimetric analyzer under an N_2 atmosphere; the cured sample (ca. 5 mg) was put in a Pt cell with a heating rate of 20 °C min^{-1} from 100 to 800 °C under a N_2 flow rate of 60 mL min^{-1}. The morphologies of the samples were examined by Field emission scanning electron microscopy (FE-SEM; JEOL JSM7610F) and also by transmission electron microscope (TEM) using a JEOL-2100 instrument at an accelerating voltage of 200 kV.

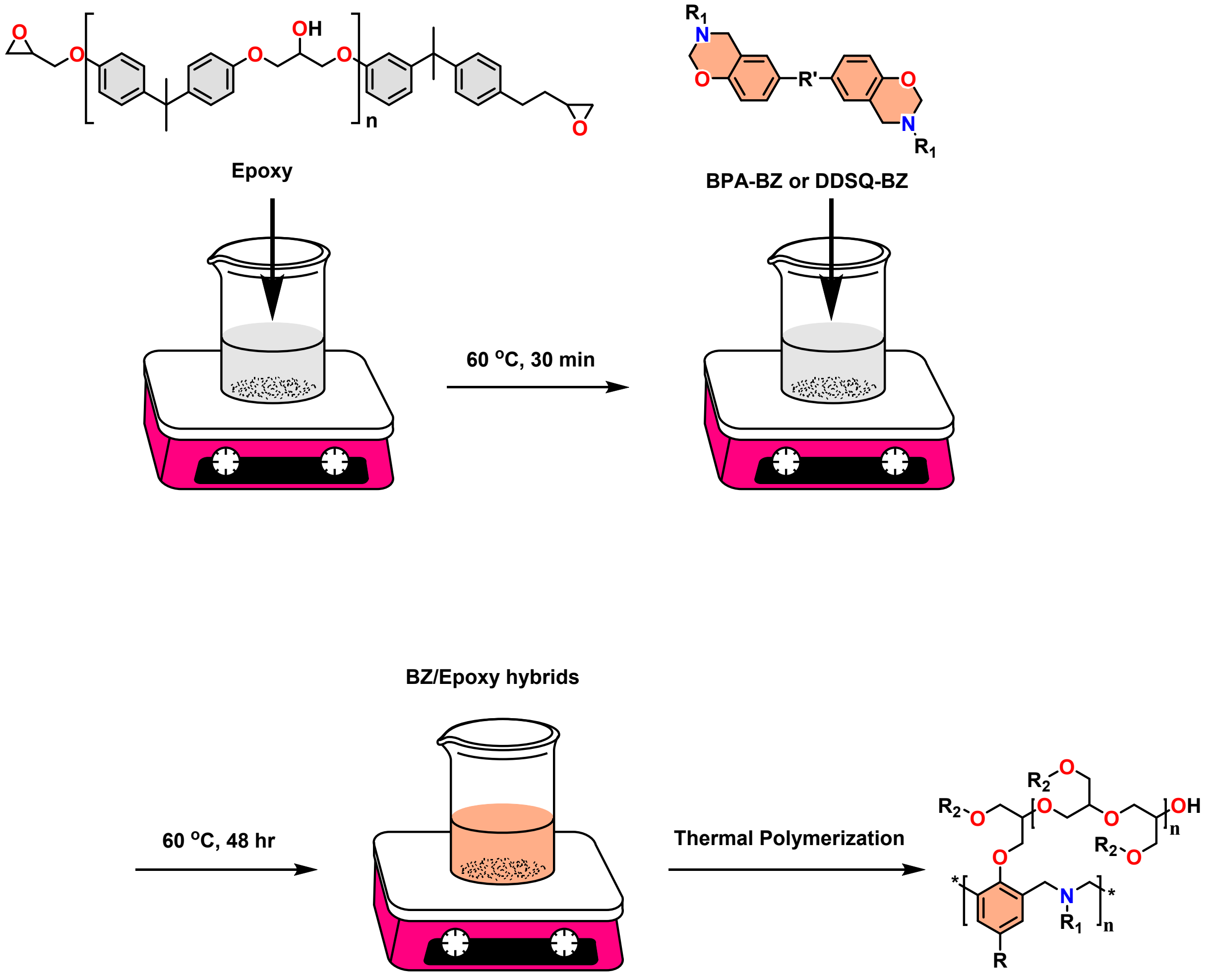

Figure S1. Schematic cartoon for preparation of benzoxazine/epoxy hybrids.
